# Effect of Epoxy Latexes on the Mechanical Behavior and Porosity Property of Cement Mortar with Different Degrees of Hydration and Polymerization

**DOI:** 10.3390/ma14030517

**Published:** 2021-01-21

**Authors:** Pengfei Li, Wei Lu, Xuehui An, Li Zhou, Sanlin Du

**Affiliations:** 1 Harbor, Waterway and Coastal Engineering, Chongqing Jiaotong University, Chongqing 400074, China; lipengfei@cqjtu.edu.cn (P.L.); lw-slime@foxmail.com (W.L.); 2 State Key Laboratory of Hydroscience and Engineering, Tsinghua University, Beijing 100084, China; zhouli18@mails.tsinghua.edu.cn; 3 Huaneng Tibet Hydropower Safety Engineering Technology Research Center, Tibet 860000, China; sanlin_d@163.com

**Keywords:** epoxy latexes modified mortar, mechanical property, porosity, degree of hydration, degree of polymerization

## Abstract

In this study, an analysis of the influence of polymer modification on the mechanical behavior, porosity, and microstructure of mortar is carried out. Epoxy latexes contents of 5%, 10%, 15%, and 20% of cement were employed in the preparation of cement mortars based on the same workability. The specimens were subjected to dry, wet, and wet–dry curing regimes. Compressive strength, flexural strength, Mercury intrusion porosimetry (MIP), and scanning electronic microscope (SEM) tests were conducted to analyze the effect of epoxy latexes on the mechanical property and porosity of modified mortars. Based on the compressive strength test results, a quantitative method was established to calculated the degree of hydration and polymerization. The results show that the mechanical behavior and porosity property of epoxy latexes modified mortar are influenced by the degree of hydration, the degree of polymerization, and the volume changing effect of mortar. The polymerization of epoxy latexes could improve the flexural strength of the mortar. The macropores of specimens tended to decrease with the increase of the degree of epoxy latexes polymerization and cement hydration. In practical engineering, it is necessary to ensure the degree of hydration and increase the polymerization rate. Thus, the wet–dry curing regime is recommended.

## 1. Introduction

Concrete is the most widely used material in China’s hydropower construction thanks to its low cost and relatively good strength in compression. Concrete structures often suffer various deteriorations caused by the ambient environment, such as frost damage, carbonation, corrosion, alkali–aggregate reaction, and so on [1]. In the construction of hydropower, the extreme environmental conditions put forward higher requirements for the properties of concrete materials. Polymer latexes modified mortar (PMM) and concrete (PMC) have been widely used as construction materials in the past decades to overcome some disadvantages of conventional cement-based materials, such as brittleness and deformation property [2,3]. As the composite material combines the advantages of polymer and concrete, PMM and PMC can effectively improve the properties of concrete structures.

With the recent development in the polymer material technologies fields, applications of polymer are more and more extensive. Innovative strengthening strategies have been developed, based on externally bonded fiber-reinforced polymer and fiber-reinforced cementitious matrix systems, to improve the capacity of existing structures in order to guarantee the safety of masonry structures [4,5,6]. Furthermore, organic–inorganic nanocomposite materials possess unique properties as new materials and compounds for academic research as well as for the development of innovative industrial applications [7,8]. In classical nanocomposites, the addition of nanofillers to a polymer matrix allows, in principle, the tailoring of physical properties. The resulting composite might exhibit improved thermal, mechanical, rheological, electrical, catalytic, fire retardancy, and optical properties [9,10,11,12,13,14,15]. The basic multifunctional feature of these nanocomposite materials makes them potentially applicable in various areas in high added-value applications such as adsorbents of toxic metal ions, smart coatings for corrosion protection, nanoscopic reactors, and so on.

In general, polymer latexes were used as a cement modifier and will form flexible polymer films in polymer-modified cement-based material [16,17]. The polymer–cement co-matrix consists of polymer films, hydration products, and cement particles, which can significantly improve the tensile strength, flexural strength, impermeability, and chemical resistance of cement-based material [18,19,20]. Various types of polymer latexes such as styrene-butadiene rubber (SBR) latex, ethylene-vinyl acetate (EVA) latex, and styrene-acrylate copolymer (SA) latex have been tested to assess their effect on the material properties at the macro-scale and micro-scale [21,22,23,24,25]. Among these polymer latexes, epoxy latexes are widely used in PMC and PMM thanks to their excellent mechanical properties, durability, and the controllable process of polymerization [26,27,28].

Numerous test programs have been conducted to study the effect of epoxy latexes on the properties of cement-based materials. The test results show that the flexural strengths and ductility of PMM and PMC are increasing significantly, while the compressive strengths are quite discrete. Aggarwal et al. [26] pointed out that the compressive strengths of the mortar increase with the epoxy–cement ratio. Ariffin et al. [29] found that, when the epoxy latexes content increased beyond 10%, the compressive strength declined. Jo [30] pointed out, in contrary reports, that the compressive strengths of the mortar decrease with the increase of epoxy latexes. Therefore, it is difficult to directly predict the modification effect of epoxy latexes from macroscopic indicators. The properties of PMM are governed by both the process of cement hydration and the epoxy polymerization [31,32]. The properties of PMM should be analyzed based on the coupled effect of hydration and polymerization. It has been reported that the addition of polymer latexes has a significant retardation effect on the cement hydration process [33,34,35]. Kong et al. [31,33] conducted experiments to investigate the effect of copolymer latexes on cement hydration and proposed the retardation mechanism of polymer latexes as two modes: chemical retardation and physical retardation. Zhang and Yan [34] investigated the retardation effect of epoxy resin on hydration kinetics at different temperatures. The test results show that the cumulative hydration heat of epoxy modified cement was relatively smaller than that of conventional cement. The retardation effect of epoxy latexes may be the reason for the scattering of the compressive strength results. Tian et al. [35] examined the microstructure formation of polyacrylate latex modified mortars and confirmed that polymer latexes will introduce big pores to cement mortars, which will increase the total porosity of PMM. The higher total porosity will lead to a decrease in the compressive strength [36]. Ma and Li pointed out that the contrary reports may be caused by the different manners in which the polymer latexes were added [37].

In general, the tensile strength, flexural strength, and deformation properties of PMM and PMC are relatively higher compared with the conventional cement-based materials [38,39]. It has been reported that the improvement of the tensile and flexural strength depends mainly on the polymer content rather than the water–cement ratio [40]. This phenomenon could be explained by the excellent tensile properties of polymer films and the forming of the polymer–cement co-matrix [17]. Polymer film will be formed within its applicable temperature and physical–chemical conditions. Some models have been proposed to illustrate the modification mechanism of the phenomenon. Ohama’s three-step model is the most widely accepted method in describing the formation process of the polymer–cement co-matrix [41]. As the model illustrated, polymer particles will be close-packed on the surfaces of cement and then coalesce into a continuous film. Several integrated models were established based on the three-step model to consider the chemical interaction between polymer and cement [42,43]. For the PMM and PMC, polymer latexes were usually used in combination with harder ones to achieve a higher rate of polymerization. Numerous studies also show that epoxy resin can be polymerized in the presence of hydroxyl ions without hardener [44,45]. The polymerization degree of epoxy has a significant influence on the mechanical properties of modified cement-based materials. Therefore, it is important to quantify the process of cement hydration and epoxy resin polymerization and clarify the effect of the coupled process on the properties of epoxy modified mortar.

In this research, polymer-modified mortars using epoxy latexes with hardener were prepared with various epoxy latexes contents and curing conditions. Compressive and flexural strength were tested to evaluate the effectiveness of modification. Mercury intrusion porosimetry (MIP) tests were conducted to analyze the effect of epoxy latexes on the porosity of modified mortar. Scanning electronic microscope (SEM) tests were used to evaluate the influence of epoxy latexes on the morphologies of the co-matrix. Based on the experimental results, a quantitative method was established to clarify the degree of cement hydration and epoxy resin polymerization. The effects of these two parameters on the properties of modified mortars were analyzed and discussed.

## 2. Materials and Methods

### 2.1. Materials

#### 2.1.1. Raw Materials

**Figure 1:**
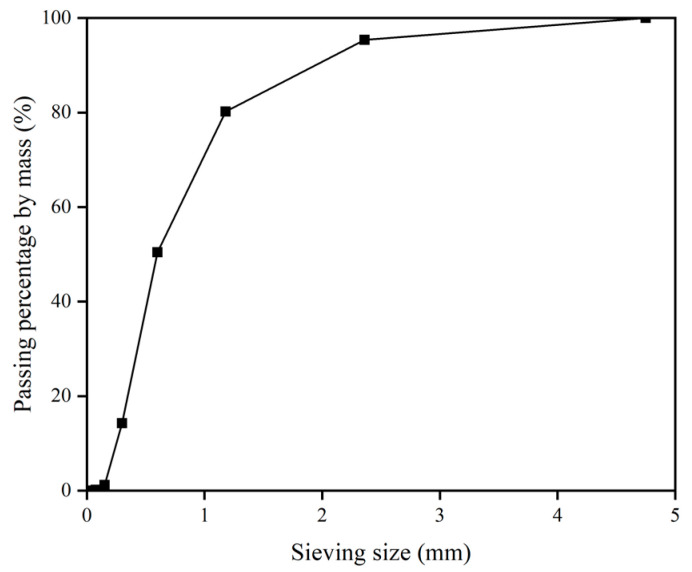
Particle size distribution curves of manufactured sand.

Type PO42.5 ordinary Portland cement with a relative density of 3080 kg/m^3^ was adopted in this research. The specific area of the cement was 390 m^2^/kg. Well-graded manufactured quartz sand with a specific gravity of 2700 kg/m^3^ was employed as fine aggregates. The particle sizes of manufactured sand range from 0.075 mm to 4.75 mm. The particle size distribution curves of manufactured sand are shown in Figure 1.

#### 2.1.2. Epoxy Latexes

**Figure 2:**
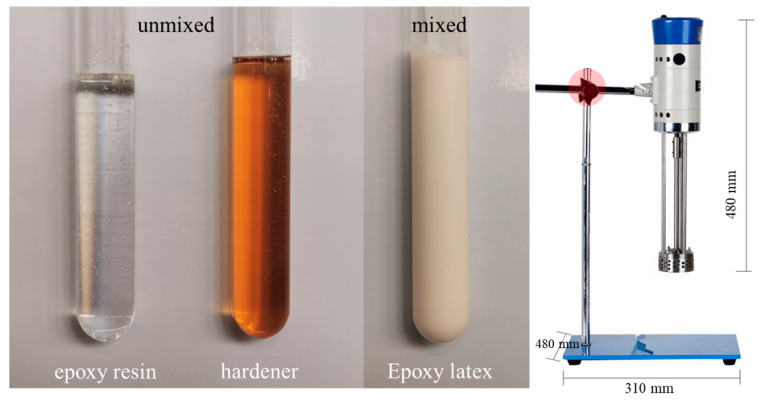
The images of epoxy latexes material of the unmixed stage and mixed stage and the high-speed shear dispersing emulsification tools.

Epoxy latexes were prepared by emulsifying epoxy resin, based on bisphenol A-type epoxy resin and curing agent in water using an emulsifier. As shown in Figure 2, the epoxy latexes material was a three-part based mixture, which consists of part A: an epoxy resin, part B: an epoxy resin hardener (composed of curing agent and emulsifier), and part C: water. In addition, the epoxy resin hardener is a self-emulsifying epoxy curing agent with the model QS-R06, which is a water-soluble curing agent formed from a variety of modified amines. The prepared epoxy resin had a density of 1.00–1.05 g/cm^3^, epoxide equivalent value of 200–300 g eq, and total solids of 99.9%. The epoxy resin hardener had a density of 1.00–1.05 g/cm^3^ and total solids of 50%.

In this investigation, a high-speed shear dispersing emulsification tool was used for mixing the epoxy latexes, as shown in Figure 2. The shearing speed of the tool was 3500 r/min during mixing. As mentioned, the epoxy latexes were composed of three parts (part A, part B, and part C). Before mixing, all components were accurately weighed according to the mix proportion, as listed in Table 1. The epoxy resin and hardener were mixed together in advance for 3 min, and then water was added for shear for another 3 min to obtain epoxy latexes. The mix proportions of epoxy latexes obtained in this research are shown in Table 1. The total solid content ratio was set and maintained at 50%.

#### 2.1.3. Mixture Proportions and Specimen Preparation

The mix proportions of epoxy latexes modified mortar were designed based on that of a self-compacting mortar. The mix proportions of self-compacting mortar were selected based on our previous research [46,47,48,49,50], which has been used in Chinese engineering. For all the mortars, the volume water/cement ratio (Vw/Vc) was set and maintained at 1.The volume ratio of sand to cement was kept constant at 1.The epoxy resin to cement (P/C) ratios of 0, 5%, 10%, 15%, and 20% were chosen to investigate the effect of various contents of epoxy latexes. In addition, a kind of polycarboxylate-based superplasticizer (SP) was used with density of 1.20 g/cm^3^ based on Chinese standard CECS 203:2006 [51], which consists of air entraining agents, water reducing agents, viscosity modifiers, and so on. The superplasticizer was the same as in the previous research [46,47,48,49,50]. With reference to JC/T 986-2018 [52], the superplasticizer was added to ensure mortars have a flowability of 200 mm. The mix proportions of epoxy latexes modified mortars are listed in Table 2. It is should be noted that the volume of water, cement, and sand was kept constant in the experiment. With the mixing of epoxy latexes, the volume of modified mortar will increase. The influence of volume increase on modified mortar is discussed in Results section. Furthermore, P/C and L/C for the modified mortar consider the ratio of dry epoxy resin to cement and epoxy latexes to cement, respectively. The hardener is incorporated to ensure that the epoxy resin is fully hardened. AD is the quality of the superplasticizer mixed in the mortar to ensure the fluidity of the self-compacting mortar. The effect of the hardener on the properties of mortar with epoxy latexes modification will be investigated in the future.

With reference to ISO 679:2009 [53], a standard laboratory mortar mixer was used to prepare the mortars. All components were accurately weighed before mixing. The fine aggregates and cement were initially blended and mixed for 1 min for all mixtures. Then, the prepared epoxy latexes, water, and superplasticizer were added slowly into the mixtures and mixed for another 2 min.

Mortar cube specimens with the size of 70.7 mm × 70.7 mm × 70.7 mm were cast for the compressive strength test and mortar prism specimens with the size of 40 mm × 40 mm × 160 mm were cast for the flexural test. After casting, the mixtures were covered with plastic sheets to avoid water evaporation. After 2 days, all specimens were demoulded and cured according to three different curing regimes.

#### 2.1.4. Curing Regimes

To investigate the coupled effect of cement hydration and epoxy resin polymerization on the properties of epoxy latexes modified mortar, dry curing, wet curing, and wet–dry curing regimes were applied for all the mortar specimens.

Dry curing: after de-moulding, the mortar specimens were transferred into a standard curing chamber and cured under the standard environmental condition at a temperature of 20 ± 1 °C and relative humidity of 60 ± 5% until the test age;

Wet curing: after de-moulding, the mortar specimens were immediately immersed in a water chamber with a temperature of 20 ± 1 °C until the test age;

Wet–dry curing: after de-moulding, the mortar specimens were immediately immersed in a water chamber with a temperature of 20 ± 1 °C for 5 d. Then, the mortar specimens were transferred into a standard curing chamber and cured under the standard environmental condition at a temperature of 20 ± 1 °C and relative humidity of 60 ± 5% until the test age.

### 2.2. Testing Procedures

#### 2.2.1. Compressive Strength

The compressive strength tests of mortar cube specimens were carried out at the ages of 7 d, 14 d, 28 d, 45 d, 60 d, and 90 d with a standard compression test machine, according to BS EN 12390 [54]. All the mortar specimens were tested up to failure with a constant load rate of 1500 ± 200 N/s. For each parameter, three specimens were prepared and the compressive strength of mortar cube specimens was calculated by the average of three specimens, as shown in Equation (1):

(1) $$f_{\mathrm{cm}}=\frac{F_{c}}{A}$$

where *f_cm_* represents compressive strength, *F_c_* is the total maximum load, and *A* is the area of the loaded surface (70.7 mm × 70.7 mm = 4998.49 mm^2^).

#### 2.2.2. Flexural Strength

The flexural strength tests of mortar prism specimens were carried out using a universal test machine at the ages of 7 d, 14 d, 28 d, 45 d, 60 d, and 90 d, according to standard ISO 679:2009 [53]. The tests were conducted under a load control mode with a constant loading rate of 50 N/s. For each parameter, three specimens were loaded up to failure. The average flexural strength of mortar prism specimens can be calculated by Equation (2):

(2) $$f_{fm}=\frac{1.5F_{f}L}{bt^{2}}$$

where *f_fm_* represents flexural strength; *F_f_* is the peak load; *L* (100 mm) is the distance between the support points; and *b* (40 mm) and *t* (40 mm) are the width and height of the cross-section of the specimens, respectively.

#### 2.2.3. MIP

The porosity and pore size distribution of mortar specimens were measured by the mercury intrusion test using a Micromeritics Poresizer model AutoPore IV 9500 (Micromeritics Instrument Corporation, Norcross, GA, USA). The mortar specimens were collected from the crushed specimens in compressive tests at the ages of 7 d, 14 d, 28 d, 45 d, 60 d, and 90 d.

#### 2.2.4. SEM

SEM tests were carried out to clarify the morphologies of epoxy latexes modified mortars. Test specimens were also collected from the crushed specimens in compressive tests at the ages of 7 d, 14 d, 28 d, 45 d, 60 d, and 90 d. All the specimens were etched with ethanol for 24 h and coated with a plastic film before the test. Magnifications ranged between 5000× and 20,000×.

## 3. Results

### 3.1. Compressive Strength

**Figure 3:**
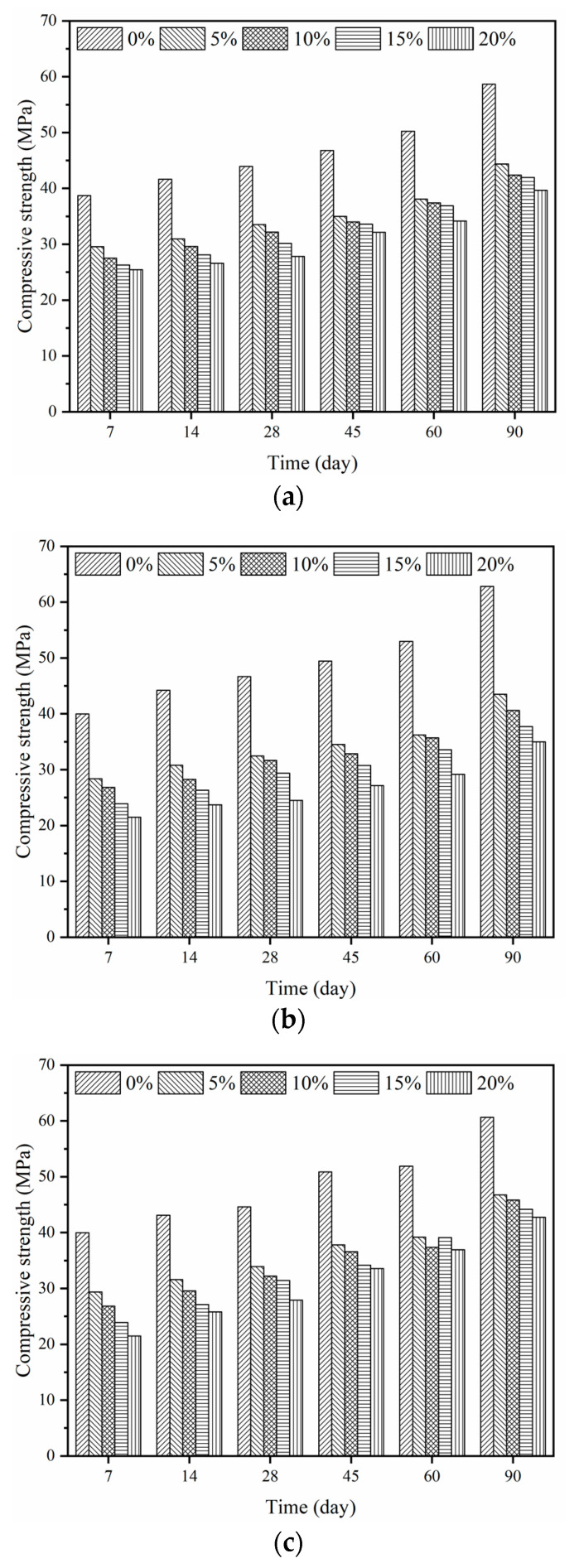
Compressive strength of epoxy latexes modified mortar: (**a**) dry curing, (**b**) wet curing, and (**c**) wet–dry curing.

The P/C ratios varied from 0 to 20%. The relationship between the epoxy latexes content and the compressive strength of mortar specimens with different curing regimes at the ages of 7 d, 14 d, 28 d, 45 d, 60 d, and 90 d is shown in Figure 3. In general, the compressive strength of epoxy modified mortars tended to decrease with the increase of the P/C ratios. In the case of dry curing, the compressive strengths of mortars with different P/C ratios had a reduction of 30–45% compared with control mortars without epoxy latexes at the age of 90 d. A similar tendency could be observed in the wet curing and wet–dry curing regimes. The result is consistent with the literature [55,56] inclusion of polymer latexes in cement mortar and concrete decreases compressive strength. The reduction in compressive strength may be caused by both the retardation effect of epoxy latexes on cement hydration and the change of the volume of mortar in unit volume, which will be discussed in the next section.

**Figure 4:**
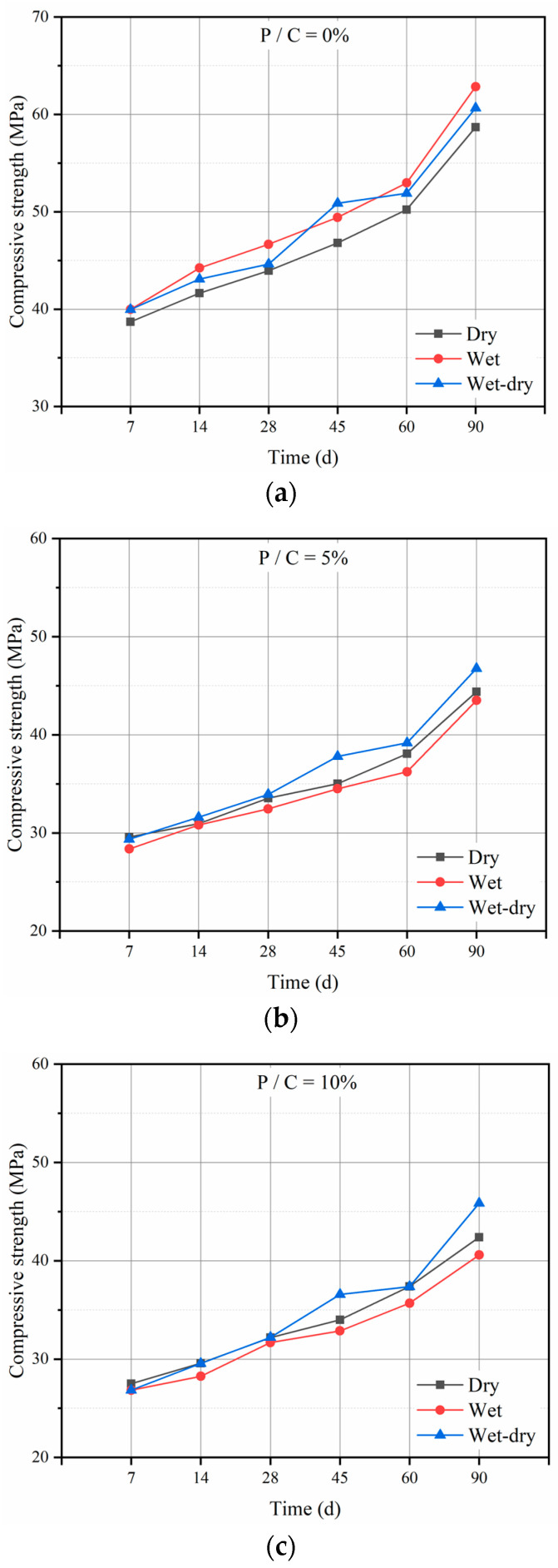

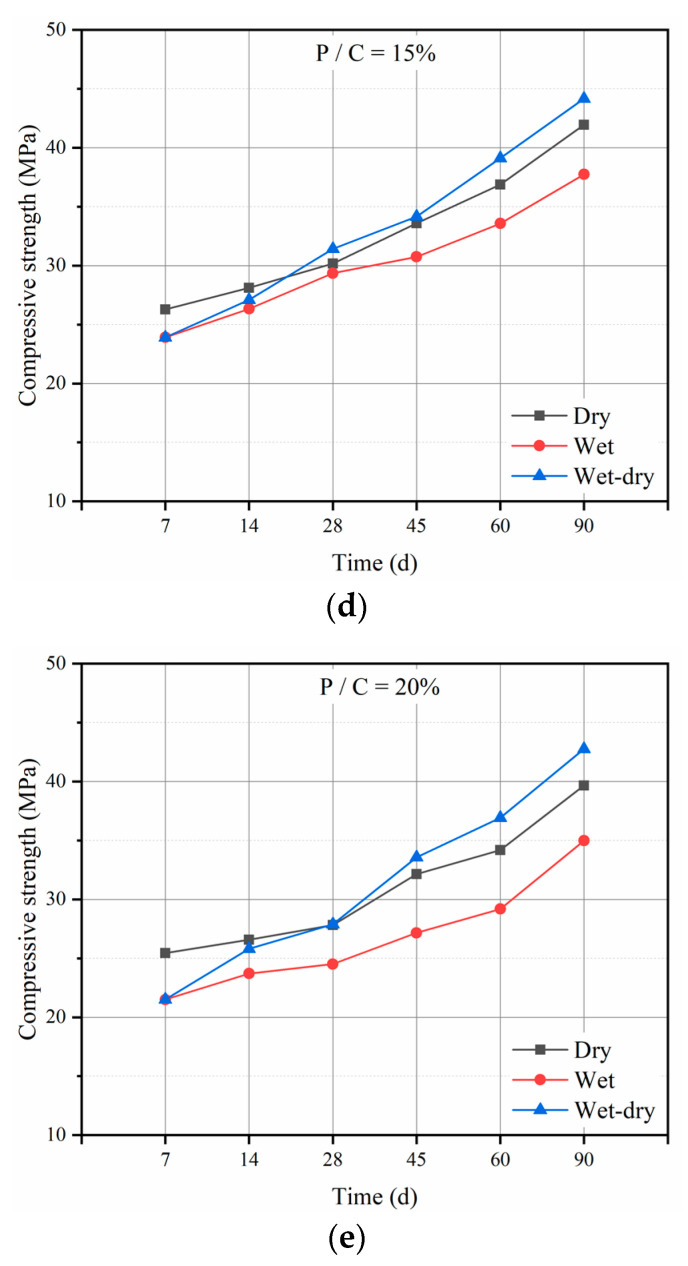
Compressive strength of epoxy latexes modified mortar under three curing regimes: (**a**) 0%, (**b**) 5%, (**c**) 10%, (**d**) 15%, and (**e**) 20%.

Figure 4 shows the development paths of compressive strength under different curing regimes. As shown in Figure 4, the compressive strength of specimens under wet–dry and dry curing regimes was higher than that of specimens under wet curing, except the control specimens. The tendency was much different from the control specimens. The distinction could be caused by the effect of epoxy resin polymerization. The epoxy resin had a much higher polymerization rate under the wet–dry and dry curing regimes. The polymer–cement co-matrix formed in mortar has a restraint effect on the expansion of mortars and has a beneficial impact on the compressive strength. The result is consistent with literature [29] showing that the wet–dry curing regime provided the optimum condition for both hydration and polymerization processes. Meanwhile, for the control specimens, wet curing regimes provide adequate water for the cement hydration and lead to a higher compressive strength.

### 3.2. Integrated Model for the Degree of Cement Hydration and Epoxy Latexes Polymerization

As mentioned, the properties of epoxy latexes modified mortar were significantly governed by both the process of cement hydration and the epoxy resin polymerization [31,32]. It is important to quantify the coupled effect and clarify the relationship between the coupled process and the mechanical properties of epoxy latexes modified mortars. In this research, the degrees of hydration and polymerization were selected as the quantitative indexes to represent the process of cement hydration and the epoxy resin polymerization, respectively. An integrated model for the degree of cement hydration and epoxy latexes polymerization was established based on the test results of compressive strength.

Neville [57] developed a simplified model regardless of the age and mix proportions to describe the relationship between the compressive strength of mortars and the cube of the gel/space ratio, as shown in Equation (3):

(3) $$f_{c}=234\cdot r^{3},$$

where *f_c_* is the compressive strength in MPa, 234 MPa represents the intrinsic strength of the hydrates gel for the type of cement, and the gel/space ratio *r* is defined as the ratio of the volume of gel and the total space available to the gel:

(4) r=khvcαvcα+w0c,

where *k_h_* is the hydrate volume expansion coefficient of ordinary Portland cement, which indicates that the products of complete hydration of 1 mL of cement will be assumed to occupy 2.06 mL gel. *v_c_* is the specific volume of cement, which denotes the volume of unit mass with a value of 0.317 cm^3^/g. *α* is the degree of hydration of cement. *w_0_* is the volume of mixing water. Finally, *c* is the mass of cement.

The validity of the model was verified by plenty of tests in the cement paste and mortar scale without polymer. In this research, new parameters will be introduced to represent the interaction between cement hydration and epoxy resin polymerization based on the model.

The degree of cement hydration is calculated by Equations (3) and (4), based on the experimentally determined compressive strengths of the control mortars. The hydration degree of cement under dry curing, wet curing, and wet–dry curing regimes was calculated by fitting the experimental results with Equations (3) and (4). Moreover, the calculated results of the degree of hydration for all specimens are listed in Table 3.

**Figure 5:**
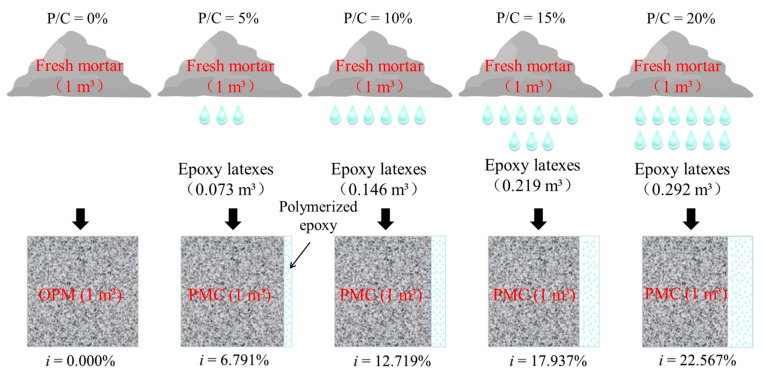
The proportion of cement mortar in the unit volume of epoxy latexes modified mortar.

It should be noted that, in this research, the mixture proportions of epoxy modified mortar were designed with a constant volume water/cement ratio (*V_w_*/*V_c_*). As shown in Figure 5, the addition of epoxy latexes will decrease the volume of mortar in unit volume. As the compressive strength of mortar specimens was affected by the volume of mortar in unit volume, the volume changing effect should be considered and the volume changing index *i* was first introduced as shown in Equation (5):

(5) $$f_{c}=\left(1-i\right)\cdot r^{3},$$

where *i* is the volume changing index and can be calculated according to Table 4.

#### 3.2.1. Retardation Effects of Epoxy Latexes

It has been reported that the addition of polymer latexes has a significant retardation effect on the cement hydration process [33]. The degree of cement hydration will decrease with the retardation effect. In this research, the effects of both the chemical retardation and the physical retardation were combined by a retardation coefficient *ξ*. The gel/space ratio in epoxy latexes modified mortar could be modified as Equations (6) and (7):

(6) r=khvcα′vcα′+w0c,

(7) $$\alpha^{\prime }=\xi \alpha ,$$

where *α’* a is the actual hydration degree of epoxy latexes modified cement mortars. *ξ* is the retardation coefficient, which indicates the retardation effect of epoxy latexes on the degree of cement hydration. For the series of control cement mortars, *ξ* = 1.

As reported before, Ohama proposed a simplified model of polymer–cement co-matrix formation wherein, with water withdrawal by cement hydration, the close-packed polymer particles on the cement hydrates coalesce into continuous films or membranes [2,58]. In the case of the wet curing regime, epoxy latexes will fail to form a continuous close-packed layer owing to the adequate capillary water. Thus, an assumption was made in this research that no polymerization reaction was produced in the case of the wet curing regime. Based on the assumption, the retardation effect coefficient of specimens under the wet curing regime could be calculated by fitting the test data by Equations (5)–(7), as listed in Table 5. The retardation effect coefficients of specimens with P/C ratios of 5%, 10%, 15%, and 20% were 0.880, 0.870, 0.865, and 0.855, respectively.

#### 3.2.2. Polymerization Effects of Epoxy Latexes

It can be seen from the test results that the polymerization of epoxy resin has a beneficial effect on the compressive strength of mortar specimens. To represent the beneficial effect, the degree of epoxy resin polymerization is introduced into the model, as shown in Equation (8):

(8) $$f_{c}=234\varphi \left(1-i\right)\cdot r^{3}$$

(9) $$\varphi =e^{\frac{\eta }{2}\cdot \frac{V_{p}}{V_{c}}}$$

where *φ* is defined as the beneficial effect of epoxy resin polymerization on the compressive strength; *η* is the degree of polymerization; and *V_p_* and *V_c_* are the volume of polymer and cement, respectively.

It has been reported that the degree of polymerization of epoxy resin has little influence on the hydration kinetics of epoxy resin-modified cement at normal temperatures [34]. Thus, the retardation effect coefficient of specimens under the dry and wet–dry curing regimes was assumed to be the same as that under wet curing regimes. Based on the calculated *ξ*, the degree of polymerization (*η*) can be calculated by fitting the experimental results through Equations (6) and (8).

**Figure 6:**
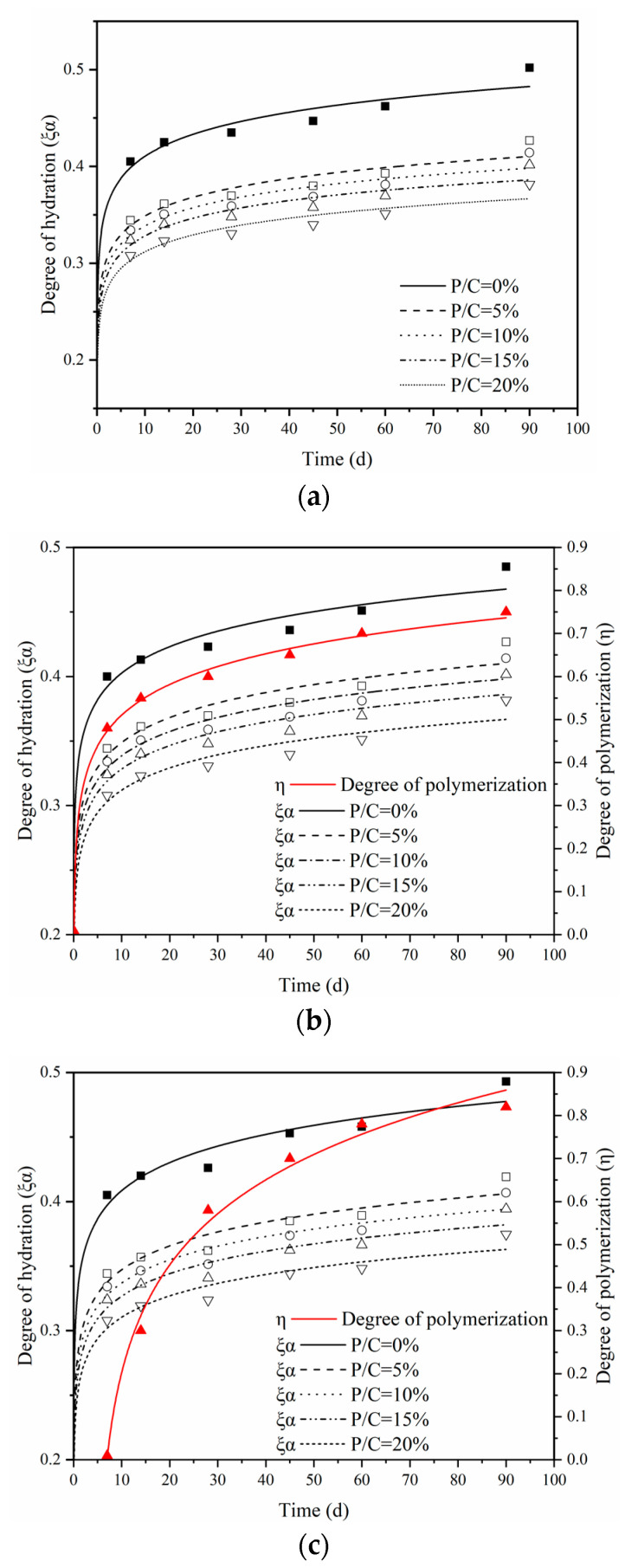
Degrees of hydration and polymerization under different curing regimes: (**a**) dry curing, (**b**) wet curing, and (**c**) wet–dry curing.

The calculated results of polymerization for all of the specimens are listed in Table 6. The comparison of the hydration degree of specimens with different P/C ratios under the dry, wet, and wet–dry curing regimes is shown in Figure 6. There is a nearly logarithmic relationship between the degree of polymerization and curing age. Both the degree of hydration and the degree of polymerization of specimens under wet–dry curing regimes were highest at later ages of 45 d, 60 d, and 90 d. The results were consistent with previous conclusions. In general, the degree of hydration decreases with the increase of the P/C ratio, and the hydration rate tends to decrease as the curing ages increase.

### 3.3. Flexural Strength

#### 3.3.1. Effect of Epoxy Latexes Modification

**Figure 7:**
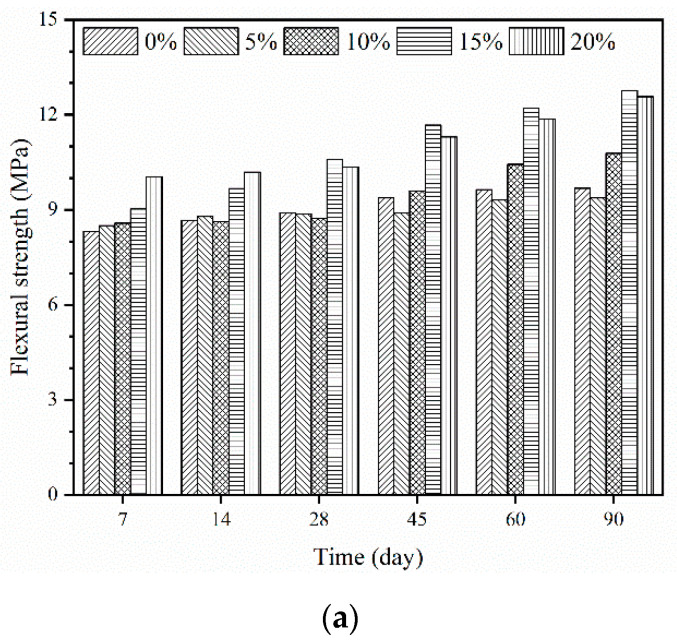

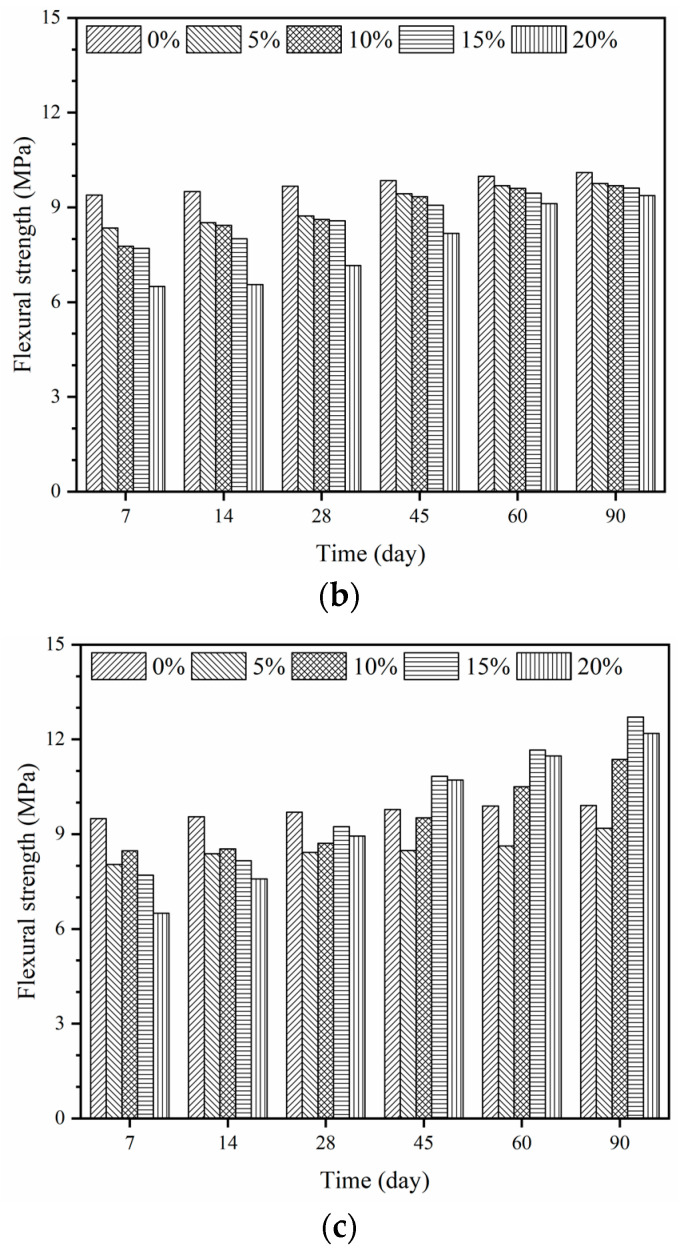
Flexural strength of epoxy latexes modified mortar: (**a**) dry curing, (**b**) wet curing, and (**c**) wet–dry curing.

An investigation of the material’s ability to resist deformation under load was done through the flexural test. Figure 7 shows the relationship between the epoxy latexes content and the flexural strength of mortar specimens with different curing regimes at the ages of 7 d, 14 d, 28 d, 45 d, 60 d, and 90 d. As shown in Figure 7a, in general, the flexural strength of epoxy modified mortars under dry curing regime tended to increase with the increase of the P/C ratios and curing ages. The flexural strength of mortar specimens with 15% and 20% epoxy content increased by approximately 30% compared with control mortars at the age of 90 d. The improvement of flexural strength could be caused by the film formation, which will increase the flexural strength of the binder matrix between the aggregate and cement hydrates. Meanwhile, for the case of wet curing, the flexural strength of epoxy latexes modified mortars was smaller than that of control mortars, as shown in Figure 7b The reduction of the flexural strength could be the low polymerization rate under the wet curing regime. Figure 7c shows the flexural strength of mortar specimens under the wet–dry curing regime; it can be seen that, at the age of 7 d and 14 d, the flexural strength of control mortars was much higher than that of epoxy latexes modified mortars. It should be noted that, for the wet–dry curing regime, mortar specimens were first immersed in a water chamber for 5 d. The low polymerization rate in the first 5 d may lead to a reduction in flexural strength. The flexural strength of epoxy latexes modified mortars increased at a relatively higher rate from 28 d until 90 d, which is consistent with literature [59] stating that the wet–dry curing regime benefited in the long term. At the age of 90 d, the flexural strength of mortars with 15% and 20% epoxy content was 28% and 23% higher, respectively, than the control mortars. It can be seen that the polymer–cement co-matrix formed in mortar has a restraint effect on the crack development of mortars and has a beneficial impact on the flexural strength.

#### 3.3.2. Effect of Hydration and Polymerization

It has been reported that the polymer–cement co-matrix formed in PMM can significantly improve the flexural strength of mortar and concrete [38,39]. It is important to clarify the relationship between the flexural and the coupled effect of cement hydration and epoxy resin polymerization. The calculated degrees of hydration and polymerization (listed in Table 3 and Table 6) were used to analyze the quantitative relation with different curing regimes. As discussed before, the compressive strength of epoxy modified mortars was influenced by the degree of hydration, the degree of polymerization, and the volume changing effect of mortar. Similarly, two influence factors were defined:

(10) $$\lambda_{1}=\left(1-i\right)\cdot \xi \alpha ,$$

(11) $$\lambda_{2}=\frac{V_{p}}{V_{c}}\cdot \eta \cdot \left(1-i\right)\cdot \xi \alpha ,$$

where *λ_1_* is the influence factor representing both the effect of cement hydration and the volume of mortar. *λ_2_* is used to represent the coupled effect of the degree of hydration, the degree of polymerization, and the volume changing effect of mortar.

**Figure 8:**
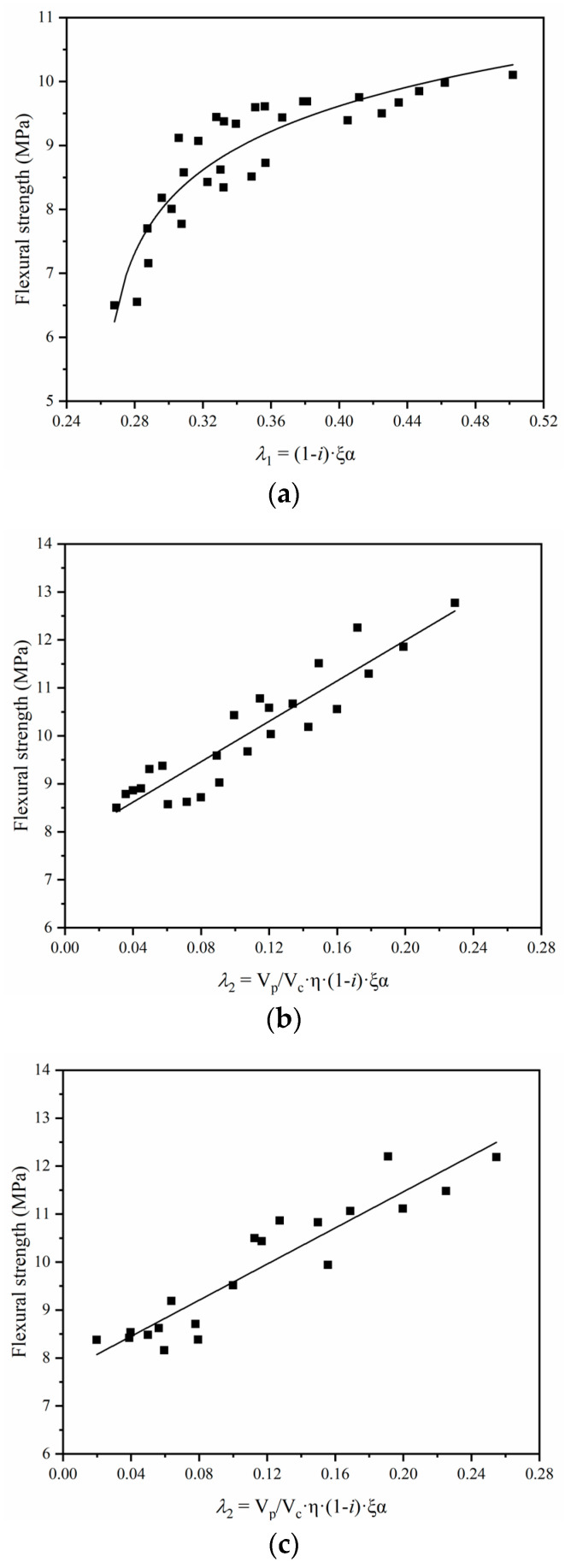
Relationship between flexural strength and *λ_1_* and *λ_2_*: (**a**) wet curing, (**b**) dry curing, and (**c**) wet–dry curing.

The relationship between the flexural strength of specimens under the wet curing regime and *λ_1_* is shown in Figure 8a. An obvious logarithmic relationship could be found. Because of the relatively lower rate of epoxy resin polymerization, the flexural strength of specimens under the wet curing regime was mainly determined by the degree of hydration. The relationship between the flexural strength of specimens under the dry and wet–dry curing regimes and *λ_2_* is shown in Figure 8b,c, respectively. The flexural strength increased approximately linearly with the increase of *λ*It can be concluded that the flexural strength of epoxy latexes modified mortar was also influenced by the degree of hydration, the degree of polymerization, and the volume changing effect of mortar. Meanwhile, the improvement of the flexural strength depends mainly on the degree of epoxy resin polymerization. It can be speculated that, for the engineering application of epoxy latexes modified mortar, the dry and wet–dry curing regimes are suitable for the development of the mechanical properties.

### 3.4. Porosity

**Figure 9:**
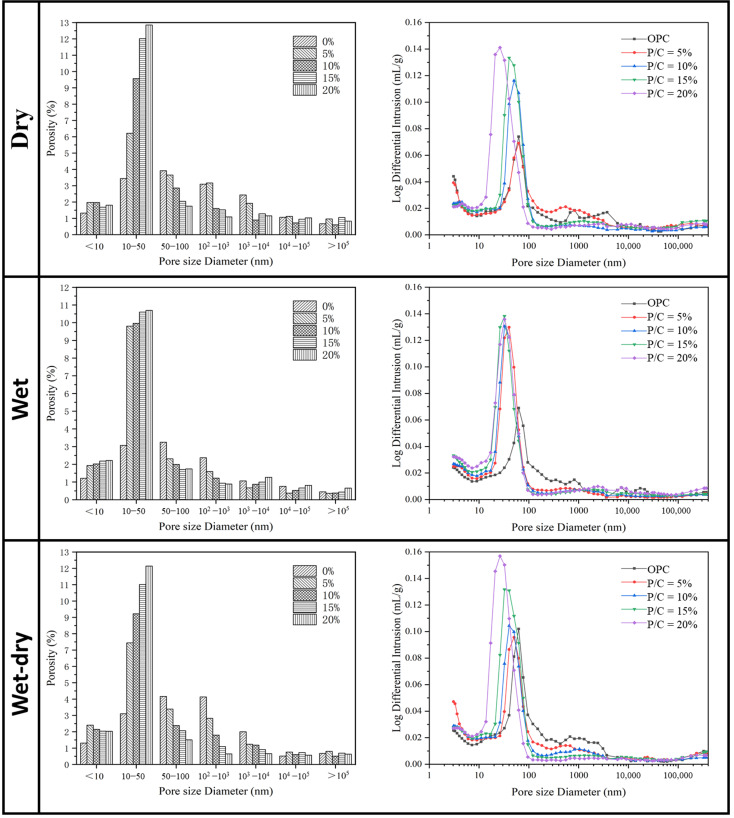
The pore structure of epoxy latexes modified mortars at 90 d under three curing regimes.

The effects of epoxy latexes modifications on pore size distributions of cement mortars determined from MIP are discussed in this section. In general, the pores in concrete can be classified as gel (d < 10 nm), transition (10 nm ≤ d < 100 nm), capillary (100 nm ≤ d < 1000 nm), and large (d ≥ 1000 nm) pores [60]. Capillary voids larger than 50 nm are referred to as macropores, whereas voids smaller than 50 nm are referred to as micropores [61]. Figure 9 shows the pore-size distributions of the mortar specimens at 90 d under the three curing regimes. As seen in Figure 9, under the dry and wet–dry curing regimes, the micropores of mortar specimens tended to increase with the increase of the P/C ratios. This tendency agrees with the results of many previous studies on polymer cement mortar [62]. Meanwhile, the peak of the curve for the epoxy latexes modified mortar specimens shifted upwards and was broader with the increase of the P/C ratio, which indicates that the capillary pores of specimens tended to increase. The increase of micropores could be caused by the retardation effect of epoxy latexes on cement hydration and the volume changing effect of mortar. Compared with the pore-size distribution under the dry and wet–dry curing regimes, the micropores of mortar specimens under wet curing regimes rapidly increased. Furthermore, the peak of the specimens shifted upwards rapidly and rapidly became broader after adding epoxy resin. It could be attributed to no polymerization reaction being produced in the case of the wet curing regime, and a large amount of free epoxy adsorbed on the surface of cement particles enhances the retardation effect of epoxy latexes on cement hydration.

**Figure 10:**
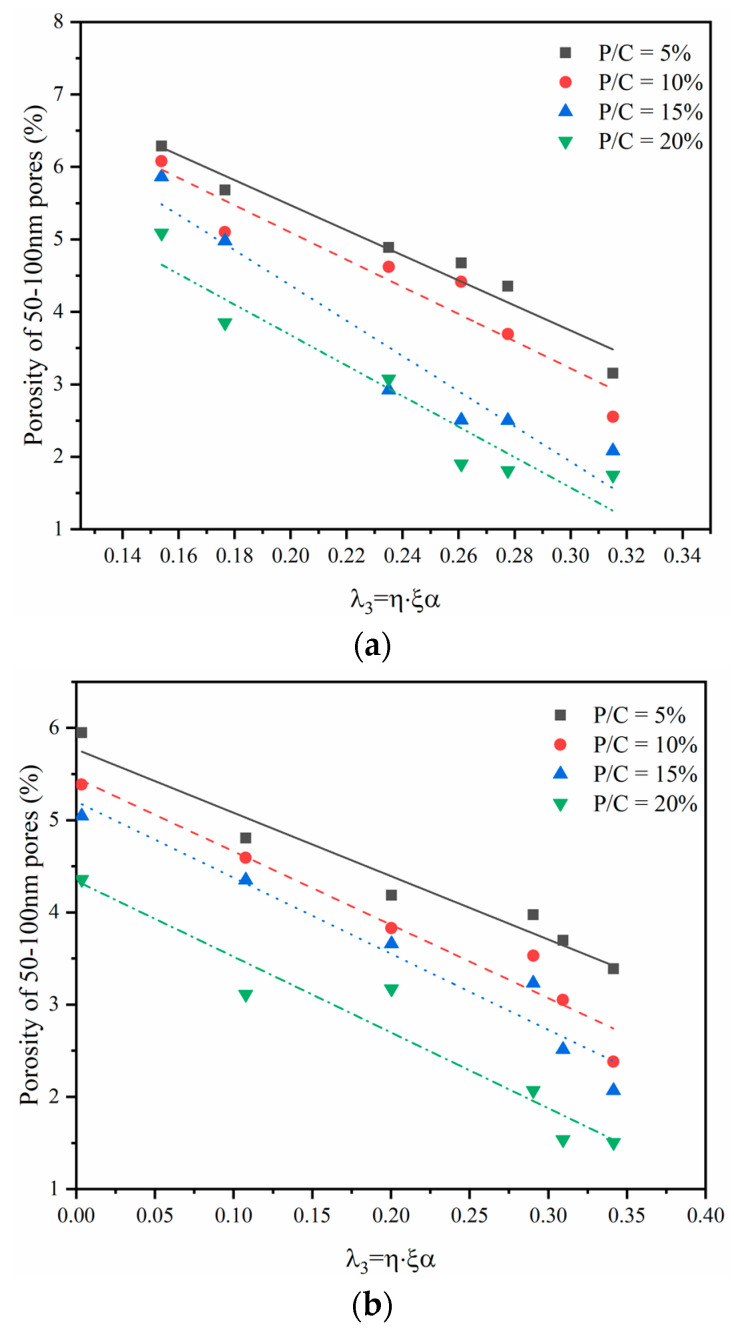
Relationship between the degree of *λ_3_* and the macropores: (**a**) dry curing and (**b**) wet–dry curing.

Similarly, an influencing factor was defined:

(12) $$\lambda_{3}=\eta \cdot \xi \alpha ,$$

where *λ_3_* is the influence factor to represent both the coupled effect of the degree of hydration and polymerization. Figure 10 shows the relationship between *λ_3_* and the macropores under the dry and wet–dry curing regimes. As seen in Figure 10, the macropores of epoxy latexes modified mortar specimens tended to decrease with the increase of the hydration products, and the polymerization of epoxy latex will also fill part of the pores. The result is consistent with literature [35] stating that more and more macropores are refined as medium capillary pores with the increase of curing ages in modified mortars. It can be concluded that the retardation effect of epoxy latexes on cement hydration leads to increased micropores, whereas macropores gradually transformed into micropores with the increase of hydration degree. The porosity reduction of the macropores is mainly determined by the cement hydrates and the filling effect of the film structure formed after epoxy latexes polymerization.

### 3.5. SEM Investigation

**Figure 11:**
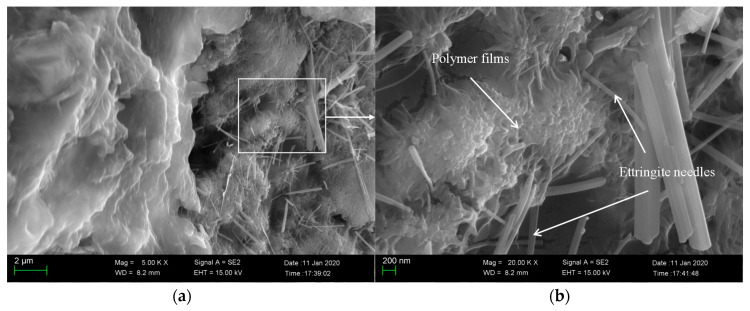
Scanning electronic microscope (SEM) image of fractured sections of epoxy latexes modified cement mortar under dry curing regimes with the addition of 15% epoxy resin at 45 d: (**a**) 5000× and (**b**) 20,000×.

**Figure 12:**
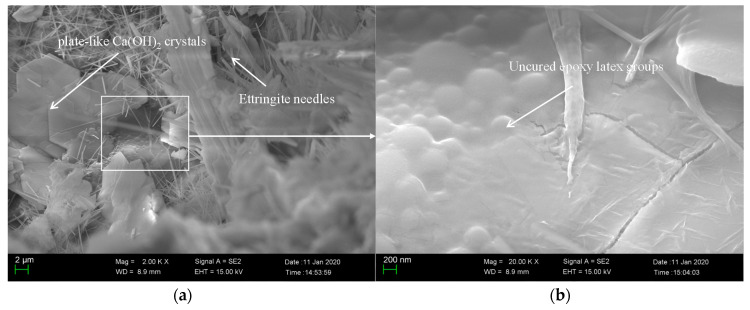
SEM image of fractured sections of epoxy latexes modified cement mortar under wet curing regimes with the addition of 15% epoxy resin at 45 d: (**a**) 5000× and (**b**) 20,000×.

**Figure 13:**
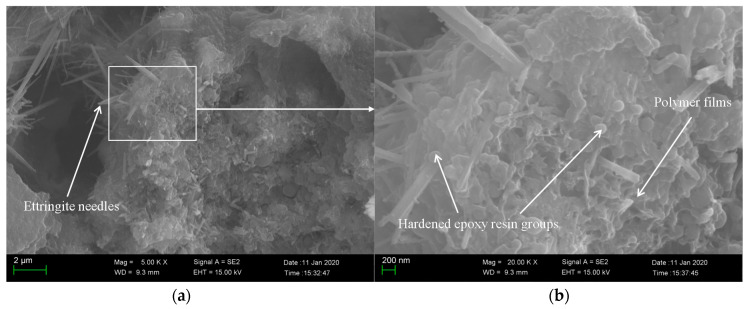
SEM image of fractured sections of epoxy latexes modified cement mortar under wet–dry curing regimes with the addition of 15% epoxy resin at 45 d: (**a**) 5000× and (**b**) 20,000×.

To understand the modification mechanism of epoxy latexes more clearly, the microstructure of the fracture surfaces of the 45 d cured modified mortar specimens under different curing regimes with the addition of 15% epoxy resin was observed by SEM. As seen in Figure 11, needle products and other cement hydrate mixtures were found in the specimens. These needle-like products are called ettringite, which is the main hydration product of cement, and it is woven together to form dense structure, which ultimately helps the high strength of cement composites [63]. At high magnifications, the polymerized epoxy resin formed as polymer films is seen in the mortar specimens, and it connects cement hydration products to enhance the interaction between crystals. As shown in Figure 12, the plate-like Ca(OH)_2_ crystals and ettringite needles produced by cement hydration could be found. However, contrary to the mortar specimens under dry curing regimes, the polymer films could not be observed clearly from the mortar specimens under wet curing regimes. It is interesting to note a large amount of unhardened epoxy resin in the mortar aggregates, which proves that no polymerization reaction was produced in the case of the wet curing regime. Meanwhile, as seen in Figure 13, the polymer network film was found in the mortar under wet–dry curing regimes, and its benefits enhance the properties of cement mortar of flexural strength. Though adding the epoxy resin leads to a decrease of the compressive strength, the polymerized epoxy resin formed as polymer films can enhance the compressive and flexural strength of the modified mortar to some degree. It could be attributed to the polymer film increasing the adhesion and bond strength of the epoxy latexes modified mortar, which heightens the ability to undergo shear forces, and reducing the risk of structural fracture.

## 4. Conclusions

In this research, polymer-modified mortars using epoxy latexes were prepared with various epoxy latexes contents and curing conditions. Compressive, flexural strength, MIP, and SEM were conducted to analyze the effect of epoxy latexes on the mechanical behavior and porosity property of cement mortar. Based on the experimental results, an integrated model for the degree of cement hydration and epoxy latexes polymerization was established. The following conclusions can be drawn from the present study:

1. The compressive strength of epoxy modified mortars tends to decrease with the increase of the P/C ratios. The compressive strength of specimens under wet–dry and dry curing regimes is higher than that of specimens under wet curing.
2. Based on the proposed model, the degree of hydration and polymerization of mortar specimens under different curing regimes can be calculated. The degree of hydration decrease with the increase of the P/C ratio, and the hydration rate tends to decrease as the curing ages increase. The compressive strength of epoxy latexes modified mortar was mainly governed by the cement hydration.
3. Epoxy latexes provide a considerable increase in the flexural strength under the dry and wet–dry curing regimes. Meanwhile, for the case of wet curing, the flexural strength of epoxy latexes modified mortars is less than that of than control mortars. The reduction of the flexural strength is caused by the low polymerization rate under the wet curing regime.
4. The flexural strength of epoxy latexes modified mortar is influenced by the degree of hydration, the degree of polymerization, and the volume changing effect of mortar. Meanwhile, the improvement of the flexural strength depends mainly on the degree of epoxy resin polymerization.
5. According to the findings of the study, the strength of epoxy latexes modified concrete is related to hydration and polymerization. In practical engineering, it is necessary to ensure the degree of hydration and increase the polymerization rate, thus the wet–dry curing regime is recommended.
6. The micropores of epoxy latexes modified mortars tend to increase with the increase of the P/C ratios. The macropores of epoxy latexes modified mortar specimens tended to decrease with the increase of the degree of epoxy latexes polymerization and cement hydration.
7. The addition of epoxy latexes is helpful to improve microstructures by forming the continuous films in cement mortar under dry and wet–dry curing regimes. Meanwhile, little continuous films can be founded under the wet curing regime owing to no polymerization of epoxy resin.

## Figures and Tables

**Table 1:** Mix proportions of epoxy latexes (wt %).

Mix Label	Epoxy Resin	Hardener	Water
epoxy emulsion	28.57	42.86	28.57

**Table 2:** Mix proportions of epoxy latexes modified mortar (kg).

Mix Label	Vw/Vc	SP%	P/C	L/C	C	S	W	AD	ER	H
Control	1.1	1.4	0%	0%	832.69	1199.80	281.46	11.66	0	0
Latex 1	1.1	1.4	5%	8.7%	832.69	1199.80	281.46	11.66	41.63	31.23
Latex 2	1.1	1.4	10%	17.4%	832.69	1199.80	281.46	11.66	83.27	62.45
Latex 3	1.1	1.4	15%	26.1%	832.69	1199.80	281.46	11.66	124.90	93.68
Latex 4	1.1	1.4	20%	34.8%	832.69	1199.80	281.46	11.66	166.54	124.90

Notes: SP% is the superplasticizer dosage, and C, S, W, AD, ER, and H are the quality of cement, sand, water, superplasticizer, dry epoxy resin, and dry hardener, respectively.

**Table 3:** The degree of hydration calculated by Equations (3) and (4).

	Curing Regimes	7 d	14 d	28 d	45 d	60 d	90 d
*α*	Wet	0.405	0.425	0.435	0.447	0.462	0.502
	Dry	0.400	0.413	0.423	0.436	0.451	0.485
	Wet–dry	0.405	0.420	0.426	0.453	0.458	0.493

**Table 4:** Reduction of cement paste in the unit volume of mortar (*i*).

P/C	5%	10%	15%	20%
*i*	6.791%	12.719%	17.937%	22.567%

**Table 5:** Experimentally determined compressive strength of specimens under the wet curing regime and the predicted results by Equations (5)–(7).

P/C	*ξ*		7 d	14 d	28 d	45 d	60 d	90 d
0%	1.000	*α*	0.405	0.425	0.435	0.447	0.462	0.502
5%	0.880	*α’ = ξα*	0.356	0.374	0.383	0.393	0.407	0.442
		Predicted (MPa)	28.10	31.21	32.82	34.79	37.31	44.37
		Experimental (MPa)	28.36	30.80	32.45	34.50	36.23	43.51
		Model absolute error	0.92%	1.33%	1.14%	0.84%	2.98%	1.98%
10%	0.870	α’=ξα	0.352	0.370	0.378	0.389	0.402	0.437
		Predicted (MPa)	26.25	29.16	30.66	32.50	34.85	41.45
		Experimental (MPa)	26.84	28.25	31.68	32.87	35.69	40.59
		Model absolute error	2.20%	3.22%	3.22%	1.13%	2.35%	2.12%
15%	0.865	α’=ξα	0.350	0.368	0.376	0.387	0.400	0.434
		Predicted (MPa)	24.31	27.00	28.39	30.09	32.28	38.38
		Experimental (MPa)	23.91	26.33	29.36	30.75	33.57	37.74
		Model absolute error	1.67%	2.54%	3.30%	2.15%	3.84%	1.70%
20%	0.855	α’ = ξα	0.346	0.363	0.372	0.382	0.395	0.429
		Predicted (MPa)	21.82	24.23	25.48	27.01	28.97	34.45
		Experimental (MPa)	21.50	23.71	24.51	27.16	29.19	34.98
		Model absolute error	1.49%	2.19%	3.96%	0.55%	0.75%	1.52%

**Table 6:** The degree of polymerization calculated by Equations (6) and (8).

	Curing Regimes	7 d	14 d	28 d	45 d	60 d	90 d
*η*	Wet	0	0	0	0	0	0
	Dry	0.450	0.500	0.650	0.700	0.720	0.760
	Wet–dry	0	0.300	0.550	0.750	0.790	0.810

## Data Availability

The data presented in this study are available on request from corresponding author.
